# Undifferentiated Carcinoma with Osteoclast-Like Giant Cells Associated with Carcinoma *In Situ* of the Main Pancreatic Duct

**DOI:** 10.70352/scrj.cr.26-0208

**Published:** 2026-06-27

**Authors:** Masanobu Taguchi, Hideki Sasanuma, Eriko Ikeda, Kazue Morishima, Hideyo Miyato, Kazuhiro Endo, Yasunaru Sakuma, Hirotoshi Kawata, Hiroharu Yamashita, Noriyoshi Fukushima, Naohiro Sata, Hironori Yamaguchi

**Affiliations:** 1 Department of Surgery, Jichi Medical University, Shimotsuke, Tochigi, Japan; 2 Department of Medicine, Division of Gastroenterology, Jichi Medical University, Shimotsuke, Tochigi, Japan; 3 Department of Pathology, Jichi Medical University, Shimotsuke, Tochigi, Japan

**Keywords:** undifferentiated carcinoma, osteoclast-like giant cells, carcinoma *in situ* (CIS), pancreas, long-term disease-free survival

## Abstract

**INTRODUCTION:**

Undifferentiated carcinoma with osteoclast-like giant cells (UC-OGC) of the pancreas is an extremely rare subtype of pancreatic malignancy, accounting for less than 1% of cases and generally associated with a poor prognosis. Its histogenesis and progression remain incompletely understood. We report a rare case in which UC-OGC appeared to be associated with carcinoma *in situ* (CIS) of the main pancreatic duct and achieved long-term disease-free survival after curative resection.

**CASE PRESENTATION:**

A 52-year-old man was referred for evaluation of a pancreatic cyst. Imaging revealed a cystic lesion with an enhancing solid component in the pancreatic tail and distal pancreatic duct dilatation. Cytological examination of pancreatic juice suggested malignancy. An open distal pancreatectomy was performed. The tumor measured 62 × 30 × 24 mm and was classified as pT3N0M0 (Stage IIA). Histopathological examination demonstrated a tumor within the main pancreatic duct extending into the surrounding parenchyma. The tumor consisted of spindle-shaped cells with numerous CD68-positive osteoclast-like multinucleated giant cells, consistent with UC-OGC. No invasive conventional ductal adenocarcinoma component was identified. CIS was identified only in the main pancreatic duct directly continuous with the UC-OGC component, whereas no CIS component was observed in the remaining main pancreatic duct or in the branch pancreatic ducts. Immunohistochemically, the tumor cells were negative for cytokeratin and positive for vimentin. Resection margins were negative, and no lymph node metastasis was detected. The patient received adjuvant chemotherapy and remained recurrence-free for 6 years and 2 months after surgery; however, recurrence subsequently developed in the liver, lymph nodes, and remnant pancreas, and he died 6 years and 4 months after the initial surgery.

**CONCLUSIONS:**

This case raises the possibility that UC-OGC may arise in continuity with CIS, suggesting a potential pathway of morphological progression from a preinvasive lesion. Although long-term survival is achievable after curative resection and adjuvant therapy, late recurrence may occur, underscoring the need for prolonged follow-up. Accumulation of further cases is required to clarify the biological behavior and optimal management of this rare entity.

## Abbreviations

CIS
: carcinoma *in situ*
PDAC
: pancreatic ductal adenocarcinoma
UC-OGC
: undifferentiated carcinoma with osteoclast-like giant cells

## INTRODUCTION

Anaplastic carcinoma of the pancreas is an extremely rare histological subtype of pancreatic cancer and is generally associated with high malignancy and a poor prognosis. In the 5th edition of the World Health Organization (WHO) Classification of Tumours, undifferentiated carcinoma is categorized into those with and without osteoclast-like giant cells.^1)^ Among these, UC-OGC of the pancreas accounts for less than 1% of all pancreatic malignancies,^2,3)^ and its pathogenesis and progression remain poorly understood. Owing to the limited number of reported cases, large-scale analyses of prognostic factors are difficult.

Here, we report a case of UC-OGC that appeared to be in morphological continuity with CIS of the main pancreatic duct and achieved long-term disease-free survival after curative resection.

## CASE PRESENTATION

**Fig. 1:**
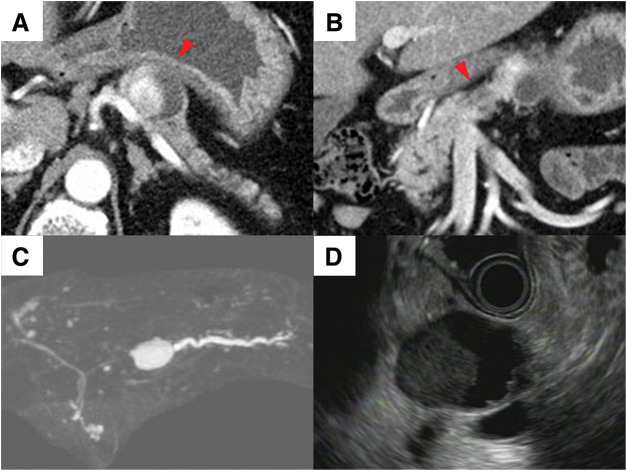
Imaging findings. (**A**) Axial contrast-enhanced CT showing a cystic lesion protruding outside the pancreas in the tail region, containing a 26-mm enhancing solid component (arrowhead). (**B**) Coronal CT showing atrophy of the pancreatic parenchyma proximal to the lesion (arrowhead). (**C**) Magnetic resonance cholangiopancreatography demonstrating a cystic lesion in the pancreatic tail with dilatation of the distal pancreatic duct. (**D**) Endoscopic ultrasonography showing a unilocular cyst with a localized solid component in the pancreatic tail.

A 52-year-old man was referred to our hospital for further evaluation and treatment of a pancreatic cyst detected at a previous institution. Laboratory examination revealed a mildly elevated serum amylase level (170 U/L), while tumor markers were within normal ranges (carcinoembryonic antigen 3.1 ng/mL, carbohydrate antigen 19-9 15 U/mL, and Duke pancreatic monoclonal antigen type 2 <25 U/mL). Contrast-enhanced CT demonstrated a cystic lesion protruding outside the pancreas in the tail region, containing a 26-mm enhancing solid component (**Fig. 1A**). The pancreatic parenchyma proximal to the lesion was atrophic (**Fig. 1B**). Magnetic resonance cholangiopancreatography revealed a cystic lesion in the pancreatic tail with dilatation of the distal pancreatic duct (**Fig. 1C**). Endoscopic ultrasonography showed a unilocular cyst with a localized solid component in the pancreatic tail (**Fig. 1D**). On endoscopic retrograde cholangiopancreatography, the main pancreatic duct distal to the cyst was not visualized. An endoscopic nasopancreatic drainage tube was placed, and serial pancreatic juice cytology revealed papillary clusters of atypical epithelial cells with nuclear irregularity, enlargement, and hyperchromasia. The findings were strongly suggestive of a malignant epithelial tumor and were classified as Class V (carcinoma). Although the histological subtype could not be determined by cytology alone, imaging suggested intraductal papillary mucinous carcinoma, PDAC associated with intraductal papillary mucinous neoplasm, or anaplastic carcinoma with cystic degeneration.

As the tumor was considered resectable, an open distal pancreatectomy was performed. Postoperatively, the patient developed chylous ascites (Clavien–Dindo grade II) and a pancreatic fistula (Clavien–Dindo grade IIIa), both of which improved with conservative management. The patient was discharged on POD 38.

**Fig. 2:**
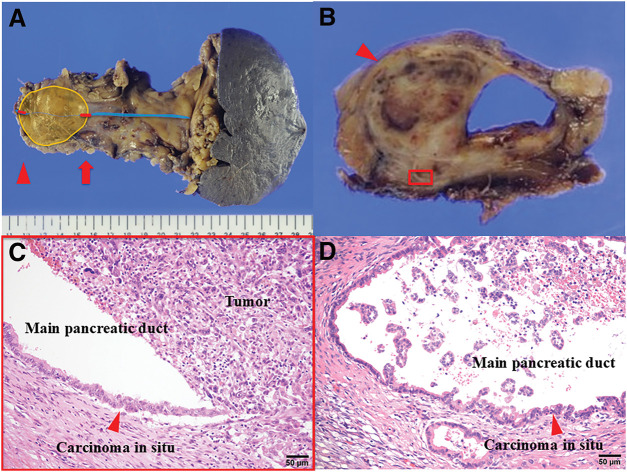
Histopathological findings. (**A**) Gross appearance of the resected specimen. The yellow-outlined area indicates the tumor; the red lines indicate the distribution of the CIS lesion, which was limited to the main pancreatic duct directly continuous with the tumor; and the blue line indicates the distal main pancreatic duct, where no CIS was identified. No CIS component was identified in the remaining background pancreas, including the branch pancreatic ducts. (**B**) Sectioned specimen showing a tumor within the main pancreatic duct (lined area) and a well-demarcated solid tumor extending into the surrounding pancreatic parenchyma (arrowhead). The tumor measured 62 × 30 × 24 mm and showed cystic change. (**C**) Microscopic findings of the lined area in (**B**) at the level indicated by the arrowhead in (**A**). CIS is observed within the main pancreatic duct and is continuous with the tumor (hematoxylin and eosin staining, ×200). (**D**) Microscopic findings of the distal boundary of the CIS component in the main pancreatic duct at the level indicated by the arrow in (**A**). No CIS component was observed beyond this boundary in the remaining main pancreatic duct or in the branch pancreatic ducts on serial whole-section examination of the background pancreas (hematoxylin and eosin staining, ×200). CIS, carcinoma *in situ*

**Fig. 3:**
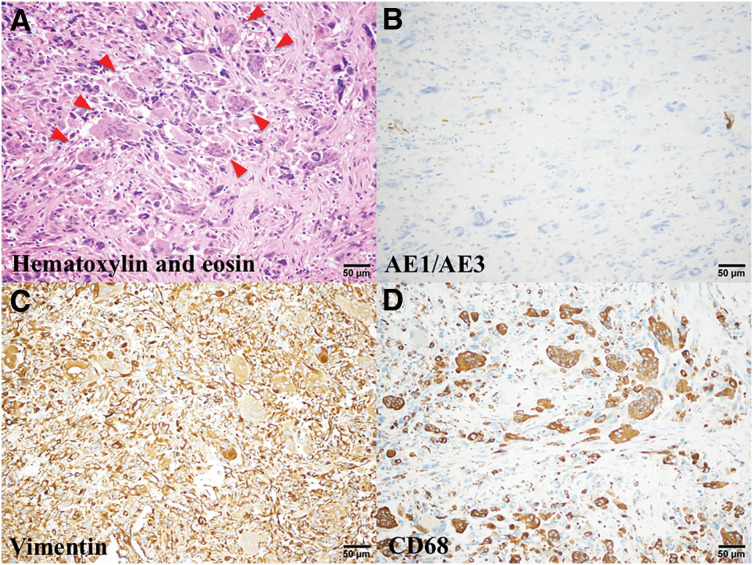
Hematoxylin and eosin staining and immunohistochemistry. (**A**) Microscopic findings of the tumor. The tumor consists of spindle-shaped cells with numerous osteoclast-like multinucleated giant cells (arrowheads) (hematoxylin and eosin staining, ×200). (**B**) Tumor cells are negative for pan-cytokeratin (AE1/AE3) (immunohistochemistry, ×200). (**C**) Tumor cells are positive for vimentin (immunohistochemistry, ×200). (**D**) Osteoclast-like multinucleated giant cells are positive for CD68 (immunohistochemistry, ×200).

Macroscopically, a solid tumor was identified within the main pancreatic duct and extended into the surrounding pancreatic parenchyma (**Fig. 2A** and **2B**). The tumor measured 62 × 30 × 24 mm and showed intratumoral hemorrhage and cystic change. It was surrounded by a fibrous capsule. Histologically, the tumor consisted of spindle-shaped cells with numerous osteoclast-like multinucleated giant cells, leading to a diagnosis of UC-OGC (**Fig. 3A**). No component of PDAC was identified on whole-section examination. CIS was identified within the main pancreatic duct, and the UC-OGC component appeared to be in direct morphological continuity with the CIS lesion (**Fig. 2C**). Serial whole sections of the resected pancreas were carefully examined to evaluate the distribution of the CIS component in the background pancreas, including both the main pancreatic duct and branch pancreatic ducts. The distribution of the CIS component is mapped in **Fig. 2A**. The CIS component was identified only in the main pancreatic duct directly continuous with the UC-OGC component, corresponding to 1 section on each side of the tumor, approximately 5 mm anterior and posterior to the tumor. The distal boundary of the CIS component in the main pancreatic duct is shown in **Fig. 2D**. No additional CIS component was identified in the remaining main pancreatic duct or in the branch pancreatic ducts throughout the examined background pancreas. These findings indicate that the CIS component was localized in continuity with the tumor rather than being diffusely distributed in the background pancreas.

Immunohistochemically, the tumor cells were negative for pan-cytokeratin (AE1/AE3) (**Fig. 3B**) and positive for vimentin (**Fig. 3C**), while the osteoclast-like giant cells were positive for CD68 (**Fig. 3D**). Immunohistochemical staining for p53 showed positivity in both the UC-OGC and CIS components. Regarding the mucin phenotype, the UC-OGC component was negative for MUC1, MUC2, and MUC5AC, and positive for MUC6. In contrast, the CIS component was weakly positive for MUC1, negative for MUC2, positive for MUC5AC, and negative for MUC6. The Ki-67 labeling index of the UC-OGC component was 64.8%. Although imaging studies suggested a cystic tumor, histopathological examination revealed cystic changes within the tumor.

Both the pancreatic resection margin and the main pancreatic duct margin were negative for tumor cells (pT3N0M0, pStage IIA, Union for International Cancer Control, 8th edition). Adjuvant chemotherapy with S-1 was administered for 7 months starting on POD 76. The patient remained recurrence-free for 6 years and 2 months after surgery; however, recurrence was later diagnosed based on contrast-enhanced CT findings showing liver lesions, lymph node enlargement, and a tumor in the remnant pancreas, together with the clinical findings of obstructive jaundice and duodenal stenosis. Endoscopic biopsy of the duodenal stenotic lesion revealed no malignant cells; however, because the stenosis was considered to have been caused by extrinsic invasion from recurrent disease, mucosal biopsy was regarded as insufficient for pathological confirmation. Thus, recurrence was diagnosed clinically and radiologically. Gastrojejunostomy and hepaticojejunostomy were performed to manage duodenal stenosis and obstructive jaundice, respectively. Gemcitabine plus nab-paclitaxel therapy was subsequently initiated. However, anemia, decreased oral intake, deterioration of the patient’s general condition, and edema developed, and chemotherapy could not be continued. Therefore, the best supportive care was selected. The patient died 6 years and 4 months after the initial surgery.

## DISCUSSION

In this case, the UC-OGC component was morphologically continuous with CIS, raising the possibility of a histogenetic relationship. Imaging and pathological findings suggested a possible process in which the tumor developed in association with CIS, caused ductal obstruction with distal dilatation and parenchymal atrophy, and subsequently extended into the pancreatic parenchyma with expansive growth and cystic change.

UC-OGC is currently classified as a histological variant of PDAC according to the WHO classification; however, its biological behavior may differ from that of conventional PDAC. The histogenesis of UC-OGC has long been debated, particularly whether it arises from pancreatic ductal epithelium or from mesenchymal cells. Morphological continuity with PanIN or PDAC,^4)^ as well as shared driver gene mutations such as *KRAS*, *TP53*, *CDKN2A*, and *SMAD4*,^5)^ now support a ductal epithelial origin. In the present case, the UC-OGC component appeared to be in direct morphological continuity with CIS, without an evident conventional PDAC component, raising the possibility that, in this case, an undifferentiated phenotype may have developed in close association with a preinvasive ductal lesion. This finding highlights the potential diversity of morphological progression in UC-OGC. To date, only 2 similar cases of UC-OGC arising directly from CIS have been reported, both demonstrating an abrupt morphological transition.^6,7)^

In this case, tumor cells were negative for pan-cytokeratin. Previous reports have described reduced epithelial marker expression and acquisition of mesenchymal markers such as vimentin during progression from PanIN3 to undifferentiated carcinoma.^4)^ Therefore, loss of cytokeratin expression in this case may reflect phenotypic transformation during progression to UC-OGC. Although morphological continuity strongly suggests a histogenetic relationship, the absence of molecular analysis precludes definitive confirmation of a clonal origin. In the present case, p53 immunostaining showed aberrant overexpression in both the UC-OGC and CIS components, which may provide supportive evidence for a possible biological relationship between the 2 components. In addition, the high Ki-67 labeling index of the UC-OGC component, at 64.8%, suggests high proliferative activity of the undifferentiated carcinoma component. Regarding the mucin phenotype, however, the UC-OGC and CIS components showed different staining patterns: the UC-OGC component was negative for MUC1, MUC2, and MUC5AC, and positive for MUC6, whereas the CIS component was weakly positive for MUC1, negative for MUC2, positive for MUC5AC, and negative for MUC6. These differences may reflect phenotypic alteration during progression from CIS to UC-OGC, including loss of epithelial differentiation and acquisition of an undifferentiated phenotype. However, immunohistochemical findings alone are insufficient to establish clonal continuity.

A major limitation of the present case is that molecular analyses, such as *KRAS* and *TP53* mutation analyses, were not performed separately for the CIS and UC-OGC components. Therefore, whether these 2 components shared common driver mutations could not be evaluated. Demonstration of identical genetic alterations in both components would have provided stronger evidence supporting clonal continuity and a direct histogenetic relationship. Conversely, distinct molecular profiles would have raised the possibility of coexistence or collision of independent lesions. Thus, the present findings should be interpreted as morphological and immunohistochemical evidence suggesting, but not proving, a histogenetic relationship between CIS and UC-OGC. Although a collision tumor cannot be entirely excluded, the lack of an intervening normal ductal epithelium and the abrupt morphological transition support a possible association between the 2 components. Therefore, this case should be regarded as a rare morphological example suggesting a possible association between CIS and UC-OGC, rather than definitive evidence of direct clonal progression.

Although UC-OGC is generally associated with poor prognosis,^3,8)^ recent clinicopathological studies have suggested that UC-OGC, especially pure UC-OGC without a ductal adenocarcinoma component, may show a better clinical course than conventional PDAC or undifferentiated carcinoma without osteoclast-like giant cells.^3,5,9,10)^ Osteoclast-like giant cells represent an important histological feature of UC-OGC. These cells are generally regarded as non-neoplastic histiocytic cells, rather than neoplastic epithelial cells, as supported by their CD68 positivity and lack of epithelial marker expression.^2,9)^ Muraki et al. reported that UC-OGC showed less frequent perineural invasion and lymph node metastasis and significantly better survival than conventional PDAC, despite larger tumor size.^9)^ In the present case, numerous osteoclast-like giant cells were observed, representing a key histopathological feature. Curative resection was achieved,^10)^ and the tumor was classified as pure UC-OGC without an adenocarcinoma component,^5)^ with no lymph node metastasis.^11)^ In addition to these favorable factors, postoperative adjuvant chemotherapy may have contributed to the prolonged disease-free survival.^12)^ However, recurrence occurred 6 years and 2 months after surgery, indicating that these potentially favorable histological features do not eliminate the risk of delayed relapse and that prolonged follow-up remains essential even after curative resection in UC-OGC.

Recent studies have reported higher expression of programmed death-ligand 1 (PD-L1) in UC-OGC compared with conventional PDAC, and PD-L1 expression may be associated with poor prognosis.^13,14)^ Moreover, immune checkpoint inhibitors have shown efficacy in metastatic UC-OGC with PD-L1 expression or high tumor mutational burden.^15,16)^ However, PD-L1 immunostaining and tumor mutational burden assessment were not performed in the present case. Therefore, the relevance of immunotherapy to this patient remains speculative. Further accumulation of molecular and immunohistochemical data is needed to clarify the potential role of immune checkpoint inhibitors in recurrent or advanced UC-OGC.

## CONCLUSIONS

This case raises the possibility that UC-OGC may arise in continuity with CIS, suggesting a potential pathway of morphological progression from a preinvasive lesion. Although long-term disease-free survival was achieved after curative resection and adjuvant chemotherapy, late recurrence occurred, highlighting the need for prolonged follow-up even after radical surgery. Given the rarity of UC-OGC, its histogenesis, biological behavior, and optimal treatment strategies remain incompletely understood. This case provides valuable insight into the origin and prognosis of UC-OGC, and may contribute to further accumulation of evidence and improved understanding of this rare entity.

## DECLARATIONS

### Use of artificial intelligence tools

The authors used an AI-based language editing tool to improve the readability of the manuscript.

The authors take full responsibility for the content.
